# A case of successful conversion from everolimus to surgical resection of a giant pancreatic neuroendocrine tumor

**DOI:** 10.1186/s40792-017-0361-8

**Published:** 2017-07-20

**Authors:** Asahi Sato, Toshihiko Masui, Nao Sankoda, Kenzo Nakano, Yuichiro Uchida, Takayuki Anazawa, Kyoichi Takaori, Yoshiya Kawaguchi, Shinji Uemoto

**Affiliations:** Division of Hepato-Biliary-Pancreatic Surgery and Transplantation, Department of Surgery, Graduate School of Medicine, Kyoto University, 54 Kawahara-cho, Shogoin. Sakyo-ku, Kyoto, 606-8507 Japan

**Keywords:** Neuroendocrine tumor, Everolimus, Sunitinib malate, ^68^Ga-DOTATOC-PET/CT

## Abstract

**Background:**

Although pancreatic neuroendocrine tumors generally have a far better prognosis relative to pancreatic cancer, the varied manifestations lead to treatment-related challenges. Everolimus therapy is generally recommended for patients with advanced pancreatic neuroendocrine tumors; however, its efficacy in a neoadjuvant setting remains unclear. Here we present a case of a giant pancreatic neuroendocrine tumor with a portal tumor thrombus that became resectable after everolimus therapy.

**Case presentation:**

A 62-year-old woman was admitted to our hospital for surgical resection of a giant pancreatic neuroendocrine tumor in the left upper abdomen. Unfortunately, she was ineligible for surgery because the tumor had extended near the hepatic hilus in the portal vein, and she was administered everolimus (10 mg/day). After 2 years of this therapy, the extent of portal vein involvement had decreased, despite the lack of significant changes in the tumor size, and the hepatic hilus became free of disease. She was subsequently referred to us for resection via distal pancreatectomy with portal vein reconstruction because the tumor had begun to grow slowly. Pathological review identified a grade 2 neuroendocrine tumor with no lymph node metastasis. The patient’s postoperative course was uneventful, and she has remained recurrence-free for 27 months, despite a lack of additional treatment.

**Conclusions:**

Our experience suggests that everolimus could be useful for neoadjuvant therapy in cases of locally advanced pancreatic neuroendocrine tumor.

## Background

Pancreatic neuroendocrine tumor (PNET) is a rare type of malignancy [1], with an estimated prevalence of 2.23/100,000 and annual onset incidence of 1.01/100,000 in Japan [2]. Although the prognosis of PNET is better than that of pancreatic cancer [3, 4], some types of PNET, such as those presenting with multiple metastases at the initial diagnosis or with advanced locoregional diseases, are difficult to cure. Recently, molecular targeting agents such as everolimus and sunitinib were found to be effective for advanced PNETs [5, 6], and the National Comprehensive Cancer Network guidelines accordingly recommend these agents for the treatment of locoregional unresectable or metastatic PNETs [7]. However, the preoperative therapeutic efficacies of these agents have not been elucidated.

In this case report, we describe the successful treatment of a giant PNET that occupied most of the pancreas and had extended via the portal vein to the level of the hepatic hilus at the time of diagnosis. Although the tumor size was not reduced significantly by everolimus, the extent of portal vein invasion was reduced enough to allowing a conversion to surgical resection. This case suggests the potential role of everolimus as a neoadjuvant agent for locally advanced PNETs.

## Case presentation

**Fig. 1:**
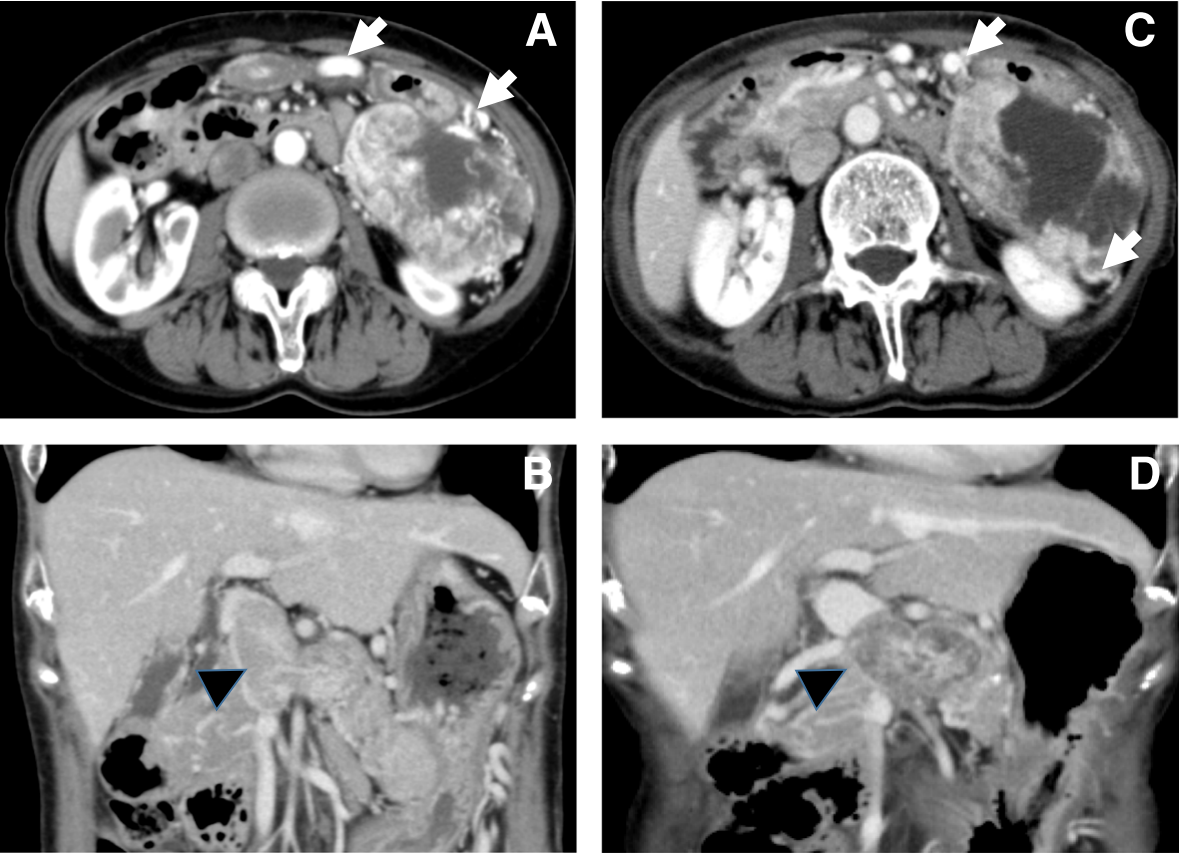
Abdominal computed tomography image showing a huge tumor in the left upper abdomen. Images were collected **a**, **b** at the time of initial diagnosis and **c**, **d** after everolimus therapy. The *arrows* indicate the collateral vessels, and the *arrowhead* indicates a tumor invasion into the portal vein

A 62-year-old woman was referred to a local hospital after receiving a positive fecal occult blood test result during an annual medical checkup. Abdominal ultrasonography incidentally revealed a giant pancreatic tumor occupying her upper left abdomen, and she was referred to our hospital for surgical resection. Abdominal contrast-enhanced computed tomography (CT) revealed a well-enhanced tumor with a maximum diameter of 13.0 cm, portal vein invasion, extension to the hepatic hilus (Fig. 1), and many collateral vessels indicative of portal stenosis. The mass inside the portal vein was also enhanced, so we considered it to be a tumor itself rather than a thrombus. No metastases were found in other organs, including the liver, lymph nodes, and lungs. A pathological endoscopic ultrasonography-guided fine needle aspiration (EUS-FNA) analysis identified the tumor as a PNET (G2). We concluded that the tumor was unresectable, given the potentially high risk of perioperative complications related to the complicated surgical techniques or liver functions. Accordingly, she began a course of everolimus therapy (10 mg/day) and was monitored on an outpatient basis at the local hospital. During the 2-year course of the therapy, the diameter of the tumor decreased slightly and the extent of the portal vein invasion decreased significantly (Fig. 1). Radiologically, the maximum diameter of the tumor was reduced to 11.8 cm. The response rate was 9.2% (stable disease in RECIST criteria). However, the tumor began to grow slowly, and she was admitted to our hospital to determine whether complete resection was feasible at that time.

**Fig. 2:**
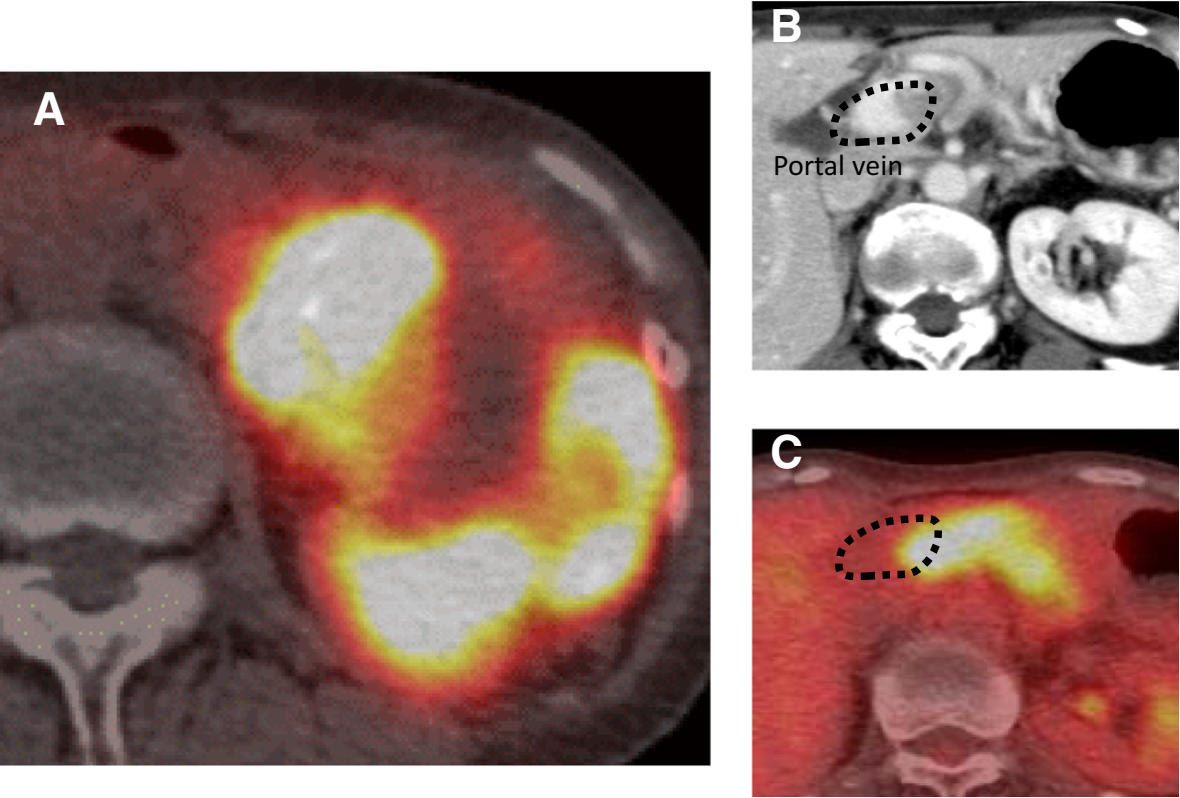
**a** ^68^Ga-DOTATOC-positron emission tomography/computed tomography indicates a high level of tracer accumulation in the tumor. However, an absence of accumulation is visible within the tumor, suggesting an effective response to everolimus. **b**, **c** The residual tumor inside the portal vein was also positive for ^68^Ga-DOTATOC

Upon admission after a 2-year everolimus course, her laboratory tests, including analyses of hormone levels such as insulin, glucagon, and gastrin, yielded unremarkable results. ^68^Ga-DOTATOC-PET/CT imaging revealed an accumulation of tracer in the tumor (Fig. 2a), but no distant metastasis. Positive accumulation was also detected in the portal vein, indicating a residual tumor inside the vessel (Fig. 2b, c).

**Fig. 3:**
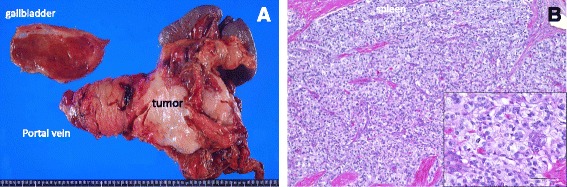
**a** Macroscopic view of the resected specimen. Although the tumor had invaded the mesentery of the transverse colon, no lymph node metastases were detected during the pathological analysis. **b** Hematoxylin and eosin staining. Hyalinosis was indicated by the *inset*

An area of low signal intensity without tracer accumulation was visible within the tumor. Endoscopic ultrasonography revealed no tumor invasion of the superior mesenteric artery or common hepatic artery (data not shown). Three weeks after withdrawing everolimus, we performed a distal pancreatectomy with portal vein reconstruction (Fig. 3a), with intraoperative mesenteric–umbilical vein shunting to keep hepatic blood flow intact. At first, we planned to harvest the left renal vein graft to put anastomosis between the superior mesenteric vein and portal vein. However, we can easily pull out the tumor from the portal vein, and macroscopic invasion seemed to be only 3 cm. Thus, we performed portal vein excision and put end-to-end anastomosis directly between the SMV and PV. The patient’s postoperative course was uneventful, and she was discharged on postoperative day 21.

**Fig. 4:**
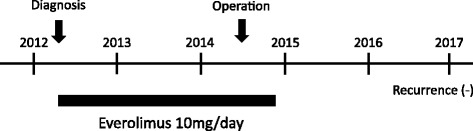
Time sequence scheme of this case

Pathologically, the tumor was composed of cells with a eosinophilic cytoplasm and “salt-and-pepper”-like nucleus. It contained liquid content inside, and microscopic analysis showed hyalinosis inside the tumor (Fig. 3b). Ki67 labeling index was 10%, indicating the tumor was a grade 2 PNET in the 2010 World Health Organization classification. Immunohistochemical analysis revealed that the tumor was positive for chromogranin A, synaptophysin, and somatostatin receptor type 2. Although the tumor had invaded the portal vein and mesentery of the transverse colon, all dissected lymph nodes were all free of involvement, and the patient did not receive any postoperative therapy. She has remained recurrence-free for more than 27 months since the resection surgery (Fig. 4).

This is a case report to describe the efficacy of preoperative everolimus therapy for locally advanced PNET. We did not initially intend to administer everolimus as a neoadjuvant therapy, following complete resection after 2 years of treatment. This suggests that the treatment plan for highly advanced locoregional PNETs must be organized individually because the tumor character is different from each other. In addition, we have to carefully estimate the therapeutic effect of the treatment for locally advanced tumor. In our case, although everolimus therapy did not appear to reduce the size of the tumor, it decreased the extent of the portal vein invasion, thus allowing us to perform surgical resection.

To the best of our knowledge, there was no English literature which suggested the effectiveness of preoperative therapy by everolimus. However, in a Japanese case report, Takano et al. reported the improvement of arterial invasion by using everolimus and somatostatin analog preoperatively [8]. Combined with our case, it might be a treatment of choice to take everolimus before resection in locally advanced PNETs with vascular invasion.

Everolimus, an inhibitor of the mammalian target of rapamycin (mTOR) signaling, was shown to effectively treat advanced PNETs in the RADIENT-3 (RAD001 in Advanced Neuroendocrine Tumors-3) study [5]. Generally, mTOR pathway abnormalities in malignancies are associated with increased aggressiveness [9], and 15% of sporadic PNETs harbor mutations in this pathway, according to an exome sequencing analysis [10]. In our patient, everolimus was especially effective for ameliorating the extent of portal vein disease, which had restricted the possibility of complete resection, suggesting the potential use of this agent as a neoadjuvant therapy for locally advanced PNETs. Additionally, our case also suggests the importance of a close follow-up of patients with unresectable locally advanced PNETs, as drug therapy may change the surgical eligibility in some cases.

In the present case, the tumor began to grow slightly after a 2-year course of everolimus therapy, leading us to speculate an adaptive response such as resistance. Although Yao et al. described the existence of an adaptive mechanism in response to everolimus [11], the acquisition of this mechanism remains under investigation. Recently, Vandamme et al. demonstrated that resistance to everolimus could be overcome using a novel phosphoinositide-3 kinase (PI3K)-Akt-mTOR inhibitor [12]. However, those results were obtained through experiments using cell lines, which differ considerably from in vivo PNETs [11]. Additional clinical experiences and the establishment of animal models that could mimic human PNETs will be needed to overcome these adaptive responses.

At present, adjuvant treatment for resected PNETs has not been established. Despite the absence of postoperative therapy, our patient has remained recurrence-free for more than 3 years after surgical resection. Our patient showed disease-free status of the lymph nodes. Hashim et al. revealed that lymph node metastasis is predictive of a poor outcome among patients with PNETs [13], and Ge et al. recently revealed the prognostic significance of lymph node metastasis regarding non-functioning PNETs [14]. Additionally, Taki et al. reported a significant difference in 5-year overall survival between patients with and without lymph node metastasis [15]. These suggest that lymph node metastasis is a risk factor for poor prognosis. In this sense, if our patient has a positive lymph node metastasis, it is reasonable to treat with adjuvant therapy. Further studies are needed to determine the indications and applications of adjuvant treatments.

## Conclusions

In our present case, we successfully resected a giant, locally advanced PNET, which was unresectable at the time of diagnosis, after a 2-year course of everolimus treatment. We have to carefully follow up treating a locally advanced tumor to estimate the possibility of resection at any time.

## Abbreviation

PNET: Pancreatic neuroendocrine tumor
